# Modelling the healthcare costs of skin cancer in South Africa

**DOI:** 10.1186/s12913-016-1364-z

**Published:** 2016-04-02

**Authors:** Louisa G. Gordon, Thomas M. Elliott, Caradee Y. Wright, Nicola Deghaye, Willie Visser

**Affiliations:** Griffith University, Menzies Health Institute Queensland, Centre for Applied Health Economics, Logan Campus, University Dr, Meadowbrook, QLD Q4131 Australia; Centre for Research Excellence in Sun and Health, Queensland University of Technology, Victoria Park Rd, Kelvin Grove Q4059 Brisbane, Australia; Environment & Health Research Unit, South African Medical Research Council and Department of Geography, Meteorology and Geoinformatics, University of Pretoria, Pretoria, South Africa; Health Economics and HIV and AIDS Research Division, University of KwaZulu-Natal, Durban, South Africa; Division of Dermatology, Department of Medicine, Stellenbosch University, Tygerberg Academic Hospital, Cape Town, South Africa

**Keywords:** Cost-of-illness, Melanoma, Squamous cell carcinoma, Basal cell carcinoma, Skin cancer

## Abstract

**Background:**

Skin cancer is a growing public health problem in South Africa due to its high ambient ultraviolet radiation environment. The purpose of this study was to estimate the annual health system costs of cutaneous melanoma, squamous cell carcinoma (SCC) and basal cell carcinoma (BCC) in South Africa, incorporating both the public and private sectors.

**Methods:**

A cost-of-illness study was used to measure the economic burden of skin cancer and a ‘bottom-up’ micro-costing approach. Clinicians provided data on the patterns of care and treatments while national costing reports and clinician fees provided cost estimates. The mean costs per melanoma and per SCC/BCC were extrapolated to estimate national costs using published incidence data and official population statistics. One-way and probabilistic sensitivity analyses were undertaken to address the uncertainty of the parameters used in the model.

**Results:**

The estimated total annual cost of treating skin cancers in South Africa were ZAR 92.4 million (2015) (or US$15.7 million). Sensitivity analyses showed that the total costs could vary between ZAR 89.7 to 94.6 million (US$15.2 to $16.1 million) when melanoma-related variables were changed and between ZAR 78.4 to 113.5 million ($13.3 to $19.3 million) when non-melanoma-related variables were changed. The primary drivers of overall costs were the cost of excisions, follow-up care, radical lymph node dissection, cryotherapy and radiation therapy.

**Conclusion:**

The cost of managing skin cancer in South Africa is sizable. Since skin cancer is largely preventable through improvements to sun-protection awareness and skin cancer prevention programs, this study highlights these healthcare resources could be used for other pressing public health problems in South Africa.

## Background

In South Africa, among the white population, there is one of the highest incidences of malignant melanoma in the world and concern for skin cancer overall has grown in recent years. The estimated yearly incidence of malignant melanoma is 4.76 per 100,000 persons overall and 19.2 per 100,000 in whites [1]. In 2009, the Western Cape of South Africa’s incidence for whites was unofficially reported as high as 69 per 100,000 population [2]. South Africans are especially susceptible to skin cancer due to their exposure to year-round high ambient solar ultraviolet radiation (UV) and latitude (22–34°S) [3]. In a population of 54 million, the racial mix within South Africa shows a diverse population, consisting of black (80.2 %), white (8.4 %), coloured (8.8 %) and Asian/Indian (2.5 %) populations [4]. “Coloured” is a Statistics South Africa non-derogatory term referring to people of mixed race in South Africa. The skin pigmentation of South African populations varies widely and although whites are most susceptible to skin cancer, skin cancer occurs in all persons regardless of their skin pigment. Squamous cell carcinoma (SCC), basal cell carcinoma (BCC) and cutaneous melanoma (CM) have the highest incidence in white people, followed by coloureds and has considerably lower incidence in both blacks and Asian/Indians [1]. However, black South Africans often present to doctors late when their melanoma has already metastasized. Research has also found that there is a high risk of developing SCC in human immunodeficiency virus (HIV)-positive South Africans [5].

The South African healthcare system has made progress towards meeting the Millennium Development Goals, although in order to reach these goals and continue an upwards trajectory, significant improvements still remain necessary [6]. The increasing incidence of skin cancers will demand larger amounts of scarce healthcare resources and will compound the stress already placed on a strained public healthcare system. The expected rising incidence of skin cancer has already been seen for CM in the Cape [7] and possibly for all cancers, including CM, in the country as a whole [8]. Targeting diseases like skin cancer which are largely preventable through better awareness and promotion of healthy behaviours among its citizens is crucial to minimizing this healthcare resource burden.

The purpose of this study was to estimate the yearly health system costs of CM, SCC and BCC in South Africa, incorporating both the public and private sectors. In doing so, it will provide a better understanding of this disease burden, the health resources used in its current management, and the potential cost savings that might arise from prevention programs [9].

## Methods

### Overview

A cost-of-illness study was used to measure the economic burden of skin cancer including malignant CM, SCC and BCC. A ‘bottom-up’ micro-costing approach was taken in order to estimate the economic burden of skin cancer diagnosis and treatment [10, 11]. The bottom-up approach identifies the patterns of care for skin cancer, assigns unit costs to each specific intervention in the care pathway and aggregates the total costs of care incurred by patients [11]. This enables extrapolation of costs on a national level based on the numbers of patients receiving each type of treatment using incidence data. We abided by all ethics principles and since we used publicly available, population level data, with no individual details, ethical clearance was not needed.

### Model

**Fig. 1:**
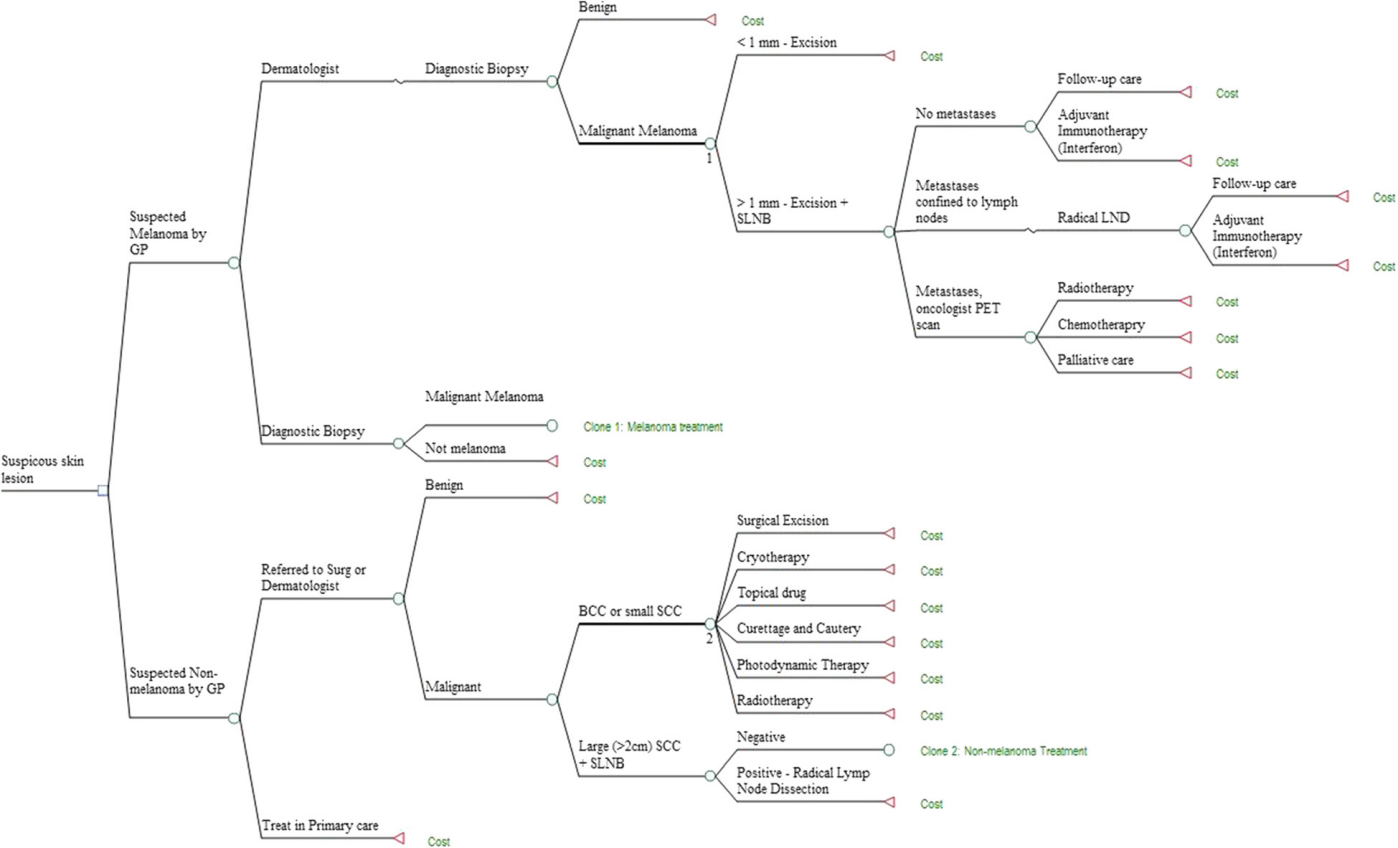
Structure of the model

A model was constructed to describe the key pathways of care in skin cancer management in South Africa using the computer program TreeAge Pro Version 2015 (TreeAge Software Inc, Massachusetts,USA). This tool is useful because it can accommodate the continuum of care from diagnosis through to treatment and follow-up. The model combines probabilities of each care pathway and their costs. In the absence of South African published skin cancer clinical guidelines, the pathways were created by a team of currently practising and experienced South African dermatologists. Consequently, these pathways reflect the current ‘real life’ patterns for private and public skin cancer care. The patterns of care aligned well with several international clinical practice guidelines for management of malignant skin cancers including those from the United States (US) National Comprehensive Cancer Network, Cancer Care Ontario, European Society for Medical Oncology and the Australian Cancer Network [12]. Separate pathways were developed for CM versus SCC and BCC (Fig. 1). The time horizon was 12 months because for most cases, treatments were completed within this period.

### Model inputs

Assumptions were necessary to complete the cost and probability values for the pathways and these are clearly stated underneath and in Tables 1 and 2.

**Table 1:** Estimated probabilities, sensitivity values and sources

Probabilities	Used in model	Sensitivity values^a^	Sources/assumptions
Melanoma			
Proportion of cases of suspected melanoma are seen in public or private setting	20 %/80 %	15 %/85 %, 25 %/75 %	Expert opinion, persons with white skin are more susceptible to skin cancers and seen in private settings
Proportion of cases seen by GP who suspects a melanoma refers to a dermatologist or surgeon	80 %	70 %, 90 %	Assumption based on convenience and high likelihood in public hospital to refer to specialist
Proportion of suspected melanoma that were malignant^b^	80 %	75 %, 85 %	Expert opinion^c^, Fong 2014 [12]
Melanoma is surgically excised	100 %	87.9 %	Expert opinion (all melanomas including advanced) Vallejo-Torres 2014 [10]
Melanoma is greater than 1 mm thick	30 %	20 %, 40 %	Expert opinion^c^
Melanoma greater than 1 mm thick has metastasized	20 %	15 %, 25 %	Expert opinion^c^
Melanoma with no metastases is treated with interferon 2b alpha	3 %	2 %, 4 %	Expert opinion^c^, Fong 2014 [12]
Melanoma greater than 1 mm thick has metastases in lymph nodes	30 %	25 %, 35 %	Expert opinion^c^, published literature - ranges from 4 to 44 %
Melanoma with lymph node metastases is treated with radical LND	100 %	–	Expert opinion^c^: All those with SLNB get RLND.
Melanoma is treated by radiotherapy	5 %	4 %, 6 %	Expert opinion^c^, most with metastases will only get palliative care, Fong 2014 [12]
Melanoma is treated with chemotherapy	10 %	5 %, 7 %	Expert opinion^c^, Fong 2014 [12]
Non-melanoma (NM)SCC or BCC			
NM is treated by a GP in the public setting	100 %	–	Expert opinion^c^, all seen first by a GP, (same for Aust. and England)
NM case is referred to a dermatologist	60 %	50 %, 70 %	Expert opinion^c^ 60 % for dermatologist or surgeon
Suspected NM is confirmed to be malignant^c^	85 %	80, 90 %	Expert opinion^c^
SCC is >2 cm diameter	10 %	8 %, 12 %	Expert opinion^c^
Large SCC is positive and surgeon treats by radical LND	20 %	15 %, 20 %	Expert opinion^c^, Fong 2014 [12]
NM is treated by:			
Surgical excision	80 %	86.0 %	Expert opinion^c^, Vallejo-Torres 2014 [10], Fong 2014 [12]
Cryotherapy	10 %	3.1 %	As above
Curette and diathermy/electrodesiccation	5 %	7.5 %	As above
Topical cream	3 %	0.5 %	As above
Photodynamic therapy	1 %	0.8 %	As above
Radiotherapy	1 %	1.7 %	As above

*LND* lymph node dissection, *GP* general practitioner, *SCC* squamous cell carcinoma, *BCC* basal cell carcinoma

^a^The sensitivity values are the high and low estimates used in the sensitivity analysis. These are based on sources in the literature or judged as plausible ranges around the best estimate used in the model base case. For probabilistic sensitivity analyses, beta distributions were assigned to probabilities to account for uncertainty

^b^Expert opinion is from practicing dermatologists and practicing doctors

^c^Lesions suspected of being Malignant are often investigated and later diagnosed as benign

**Table 2:** Estimated unit costs (2014/2015 South African Rand ZAR and USD) and sources

	Private sector	Public sector	Weighted cost^a^(ZAR)	Weighted cost^a^(USD)	Source
Cost of a dermatologist or surgeon visit	458	268	298	51	UPFS tariffs #1012 + #1010 (facility fee level 2)
Cost of a GP visit	335	158	186	32	UPFS tariff #1011 + #1010 (facility fee level 2), private: review of invoices
Cost of a diagnostic biopsy (includes immunohistology)	750	593	618	105	UPFS tariff #1112 + #1110 (facility fee level 2), private: R750 dxbiopsy + excision
Cost of excision	4643	593	1241^b^	211	UPFS tariff #1112 + #1110 (facility fee level 2), private: review of invoices
Cost of an excision with reconstructive surgery	4433	2091	2466	418	UPFS tariff #1612 cosmetic surgery cat A -specialist \practitioner + 75 facility fee^b^
Cost of cryotherapy	1257	593	699	119	UPFS tariff #1112 + #1110 (facility fee level 2),^b^
Cost of topical cream	548	548	548	93	Master procurement list - Imiquimod 5 % 12 sachets #180346230
Cost of photodynamic therapy	2514	1186	1399	237	UPFS tariff #1112 + #1110 (facility fee level 2), need 2 sessions^b^
Cost of curettage and cautery	1257	593	699	119	UPFS tariff #1112 + #1110 (facility fee level 2),^b^
Cost of radiotherapy - whole course	62334	29403	34672	5884	Annex N for Radiology - MR planning for radiotherapy course (oncol radiotherapy not available) Have used Aust. Price $7325 for whole course^c^
Cost of chemotherapy - whole course	31185	31185	31185	5292	Dacarbazine was not on the procurement list therefore price is based on fotemustine, AU $7769 per course^c^
Cost of treatment for SCC or BCC in primary care by GP	1018	480	566	96	UPFS tariff #1112 + #1010 (facility fee level 2),^b^
Cost of adjuvant immunotherapy with interferon - interferon alpha, 1 syringe injection	367	367	367	62	Master procurement list interferon alpha 2b syringe 9 MIU/0.5 mL #180348820 - daily injection for 10–24 weeks, then 3 ×/week for up to 6 mths
Cost of PET scan	11195	4224	5339	906	UPFS tariff #1952 + #1950 (facility fee level 2), private: review of invoices
Cost of a radical lymph node dissection	6632	593	1559	265	UPFS tariff #1112 + #1010 (facility fee level 2),
Cost of sentinel lymph node biopsy	750	593	618	105	UPFS tariff #1112 + #1010 (facility fee level 2),^b^
Cost of follow up	1694	158	404	69	UPFS tariff #1031 + #1010 (facility fee level 2)
Cost of hospitalisation - 1 night for melanoma with SLNB	2105	993	1171	199	UPFS tariff #0612 + #0610 (facility fee level 2) same as day patient #0663^b^

*UPFS* Uniform Patient Fee Schedule, *GP* general practitioner, *SLNB* sentinel lymph node biopsy, *PET*positron emission tomography, *SCC* squamous cell carcinoma, *BCC* basal cell carcinoma, *MR* magnetic radiation

^a^Weighting based on 16 % private and 84 % public [23]. Sensitivity analyses are based on the private and public sector values

^b^In the absence of a private fee, it was assumed a 2.12 to 1.00 ratio of private to public based on the cost of a GP visit differential between private and public sectors

^c^Converted to ZAR by http://eppi.ioe.ac.uk/costconversion/default.aspx

Note: Sensitivity values were created with a 15 % change in the public sector probability when weighting the cost. (low = 84 %*0.85;high = 84 %*1.15). For probabilistic sensitivity analyses, gamma distributions were assigned to costs to account for uncertainty

#### Probabilities

A thorough literature search was performed to identify published studies in the medical literature and organisational websites that report on the patterns of skin cancer care in South Africa. There were no data on the probabilities of different treatment options typically used in South Africa, and we relied on two clinical experts for these estimates. The estimates were varied around plausible ranges and tested in sensitivity analyses. Table 1 provides the values used in the model, their ranges tested and sources. For simplicity, and due to data restrictions, a number of assumptions were necessary. These included: a person would only develop one skin cancer in the 12-month period; a person who received sentinel lymph node dissection would have an overnight stay in hospital prior to the procedure; all suspected melanomas had a diagnostic biopsy; treatments were all one-offs; benign lesions received no treatment; and no re-treatments occurred.

#### Costs

Healthcare resource usage was identified through the treatment descriptions. The main components of costs in the model were: initial and follow-up consultations by general practitioners or specialists, pathology and treatment alternatives for example, surgical excisions, topical creams, photodynamic therapy etc. In the public health system, most patients will be seen by a general practitioner at the primary health care level and subsequently treated in a secondary level public hospital. Costs for public hospital services were derived from the National Public Hospital Tariff schedule [13]. Costs for medicines such as imiquimod 5 % cream and interferon 2b were sourced from the South African Master Procurement List [14]. Costs for patients in the private system were derived from fees charged by private practitioners with recent invoices reviewed as evidence of these. Although charges for services are not strictly economic costs, they do represent a monetary value of the present care provided to patients for skin cancer. Costs which were not in the above resources were converted into Rand from costs in the Australian healthcare system (Table 2). We combined the private and public costs into weighted averages for each item (Table 2) in recognition of the 84 % public and 16 % private system split in South Africa [8].

### Analyses

All costs are presented in 2014/15 South African Rand (ZAR) and US dollars ($). The currency exchange rate was 1 ZAR = 0.1697 US using the Cochrane cost converter at http://eppi.ioe.ac.uk/costconversion/default.aspx which inflates and converts currencies simultaneously using purchasing power parities. The model aggregated all probabilities and costs to derive the mean cost per person for malignant CM or SCC/BCC. One-way sensitivity analysis was performed on all estimates to determine the reasonable variation in values where uncertainty and variation in clinical practice exists. Probability distributions were assigned to parameters with the highest amount of uncertainty and a probabilistic sensitivity analysis was performed to calculate the mean cost of CM and SCC/BCC skin cancer.

The mean costs per melanoma and per SCC/BCC were extrapolated to estimate national costs using published incidence data from 2000 to 2004 [1] and official population statistics [4]. Notifications of all histologically-confirmed cases of SCC, BCC and malignant CM are received by the South African National Cancer Registry (NCR). The NCR is the largest and most representative cancer registry in South Africa. Reporting processes are robust with quality-assurance measures in place [8]. However, between 2005 and 2011, this voluntary registry did not receive all private hospital registrations (up to 16 % of all patients). The impact of this was the underestimation of the true number of cases. A study by Singh et al. [8] has quantified this gap in case numbers for cancers overall (4 %) and our estimates were adjusted accordingly. The Singh [8] study did not include non-melanoma skin cancers, however it is assumed the reporting issue for these cancers would be equivalent. The ethnic group incidences sourced from Norval et al. [1] were not complete. The ethnic group of patients were unspecified in 93 % of cases. The study used a hot-deck imputation method to allocate surnames into ethnic groups, although a large proportion of surnames could not be allocated. A sub-group ‘unknown race’ was created to represent this population.

Benign skin lesions, suspected to be malignant, are often included in estimates of skin cancer cost [10, 15]. We also modelled the diagnostic or ‘screening’ costs of skin cancers where the medical consultation involved investigation of a suspicious skin lesion that was subsequently found to be benign and required no further treatment. The proportion of skin cancer investigations which were benign was sourced through the expert opinion of practising medical staff, although it is acknowledged the accuracy of the number of SCCs and BCCs treated by GPs is unknown.

## Results

Our model predicted that the annual total cost of treating skin cancers in South Africa were ZAR 92.4 million ($15.7 million). This assumes all those diagnosed are treated as per local clinical practice however, despite some anecdotal evidence that loss to follow up may be high in the public sector in South Africa. The estimated costs were ZAR 81.6 million ($13.8 million) for non-melanoma skin cancer and ZAR 10.8 million ($1.8 million) for CM (Table 3). When lesions suspected to be skin cancers, but were diagnosed as benign, were considered, these additional costs were ZAR 45.1 million ($7.7 million). The total skin cancer costs per ethnic group were highest for whites (ZAR 30.5 million ($5.2 million) or 33.0 %), coloureds (ZAR 9.69 million ($1.6 million) or 10.5 %), black Africans (ZAR 7.84 million ($1.3 million) or 8.5 %) and lowest for Asians/Indians (ZAR 0.44 million ($0.1 million) or 0.5 %) (Table 3). The remaining ZAR 44.0 million ($7.5 million) (47.6 %) was attributed to persons of ‘unknown race’. The validity of the cost distribution across the ethnic subgroups is diminished due to the ‘unknown race’ subgroup representing almost half the total costs. Consequently, the estimates are grossly underestimated and are a limitation of the current data quality in South Africa.

**Table 3:** Estimates of national costs of skin cancer (2014/15 ZAR, $US)

	Melanoma	BCC and SCC	Total
Mean costs per suspected case	ZAR 3566 $605	ZAR 2154 $366	-
Mean costs per diagnosed case	ZAR 4197 $712	ZAR 2767 $470	-
	<1 mm ZAR 2931 $497		
	>1 mm ZAR 7149 $1213		
Incidence per 100,000^a^	4.77	54.6	59.4
	million	million	million
Total costs	ZAR 10.80 $1.8	ZAR 81.60 $13.8	ZAR 92.40 $15.7
By racial group			
Blacks	ZAR 2.09 $0.4	ZAR 5.76 $1.0	ZAR 7.84 $1.3
Asians/indians	ZAR 0.05 $0.0	ZAR 0.38 $0.1	ZAR 0.44 $0.1
Coloureds	ZAR 1.04 $0.2	ZAR 8.66 $1.5	ZAR 9.69 $1.6
Whites	ZAR 3.67 $0.6	ZAR 26.82 $4.6	ZAR 30.49 $5.2
Unknown race	ZAR 3.96 $0.7	ZAR 40.06 $6.8	ZAR 44.02 $7.5
By Gender			
Men	ZAR 6.10 $1.0	ZAR 54.71 $9.3	ZAR 60.81 $10.3
Women	ZAR 4.70 $0.8	ZAR 26.96 $4.6	ZAR 31.67 $5.4

*SCC* squamous cell carcinoma, *BCC* basal cell carcinoma, *ZAR* South African Rand

^a^Norval 2014 [1] - Adjusted by 1.04 for under-reporting as per Singh 2015 [8]

Sensitivity analyses showed that the annual cost of skin cancers could vary between ZAR 89.7 to ZAR 94.6 million ($15.2 to $16.1 million) when melanoma-related variables were changed and between ZAR 78.4 to ZAR 113.5 million ($13.3 to $19.3 million) when non-melanoma-related variables were changed. The primary driver in costs were the costs of follow-up and investigations, cost of excision and histopathology and the proportion of public patients, melanoma >1 mm and non-melanoma referrals.

## Discussion

This is the first cost-of-illness study performed on skin cancer in South Africa. The total cost of treating skin cancer was estimated to be in the vicinity of ZAR 92.4 million ($15.7 million) a year in South Africa. This is likely to be underestimated due to not all BCC being monitored throughout South Africa and also excludes the costs associated with potential life lost and lost productivity, as included in other studies [16]. However, putting this finding into perspective, if skin cancer was prevented and the funds currently spent on diagnosis and treatment were redeployed, ZAR 92.4 million ($15.7 million) could provide two doses of human papilloma virus (HPV) vaccine to 305,000 girls in public schools (this translates to 67 % coverage of Grade 4 girls aged 9 and older, based on 2014 data)^1^. Cost-of-illness studies are limited for health policy decisions because they do not provide information on cost-effectiveness and therefore, cannot guide decisions about the wisest choice of interventions to be provided. However, skin cancer is preventable if sun exposure is not excessive and therefore cost studies are important and useful for raising awareness for preventable diseases, and they illustrate the costs that may be averted through prevention programs. Many studies have previously shown that prevention initiatives such as sunscreen promotion, educational programs, and multifaceted programs are cost-effective [17–22]. These programs have been effective in the context of organisational settings such as educational locations as well as workplaces and sporting bodies and at systemic levels through mass media campaigns.

Skin cancer is known to be caused by solar ultraviolet radiation and sun protection strategies are well-established in primary prevention. Preventing skin cancer through clothing, hats, sunscreen and seeking shade when outdoors, are known to reduce skin cancer development. In a strained healthcare system, understanding the impact of averting skin cancers emphasizes the economic importance of skin cancer prevention as a way of not only promoting health but freeing health resources for other conditions. Due to the lack of recent cancer statistics and monitoring systems, the study highlights the need for efficient surveillance and data capturing, increased research, improved awareness and informed prevention of skin cancer.

South Africa has a pluralistic health system, where the public and private sectors have radically different resources . Approximately 80 % of the population is served by an under-resourced and severely strained public health system where treatment is provided free of charge [23]. The remaining 20 % of the population (mostly those who are formally employed) receives world-class health care from private healthcare providers, through private medical insurance. Health resources are heavily skewed towards the private sector, which serves the minority of the country’s population. There are racial differences in health care utilisation: 60 % of white and coloured adults visit a health professional in a year, of which 81 % were to a private facility, compared to 44 % of black African adults, of which 34 % were to private facilities [24]. The lower rates of black South Africans seeking medical care are explained, in part, by high travel costs to attend health care, out-of-pocket cost burden, long queues, perceived disrespectful treatment by facility staff, medicine stock-outs, perceived ineffective care and a preference to see traditional healers [25]. This behaviour has affected the presentation of melanoma in black South Africans with nodal disease occurring in more than one third of patients at their initial visit and 15 % already having disseminated metastatic disease [26, 27]. The lack of concern for skin problems relative to other serious health worries for people with HIV/AIDS may also be an additional reason for late presentation. Because black South Africans are not immune to skin cancers and because of high HIV prevalence, sun protection messages remain important [28].

A recent systematic review of studies reporting national costs of skin cancers and cost-effectiveness studies of prevention programs indicates the significant economic burden of skin cancer around the world [29]. The review found that in 16 studies, as a ratio to the population size of the country, the highest annual direct health system costs, is felt in Australia, New Zealand, Sweden and Denmark. If the results of the present analysis were available at the time of the review, it would have showed that South Africa was lowest for melanoma burden but higher than Brazil for non-melanoma cost burden (as a ratio of 2013 euros to population size) [29].

This analysis is limited because it relies on simplifying assumptions and on limited expert opinion for the treatment probabilities in the model rather than on large observational studies. Although not ideal, such data is not available in South Africa. Nevertheless, our estimates are ‘real life’, contemporary and the types and frequencies of treatments are comparable to those of the general international literature and guidelines for skin cancer [12]. For example, excision is clearly the preferred and dominant approach for non-melanoma skin cancer and melanoma. In these types of modelling studies, gaps commonly occur and reliance on expert opinion for these estimates is often necessary [30]. Treatments of skin cancers are also changing. New targeted therapies for advanced melanoma (e.g., dabrafenib, ipilimumab) are now available in other countries and are very expensive. If these are accepted for use in South Africa, the estimated cost of ZAR 10.8 million (US$1.6 million) for treating CM will be even higher. Lastly, the incidence data used to extrapolate the total costs were calculated using data from 2000 to 2004, and may no longer be accurate, and under-reporting is strongly suspected for BCCs because health care services do not fully cover all areas of South Africa [1].

**Fig. 2:**
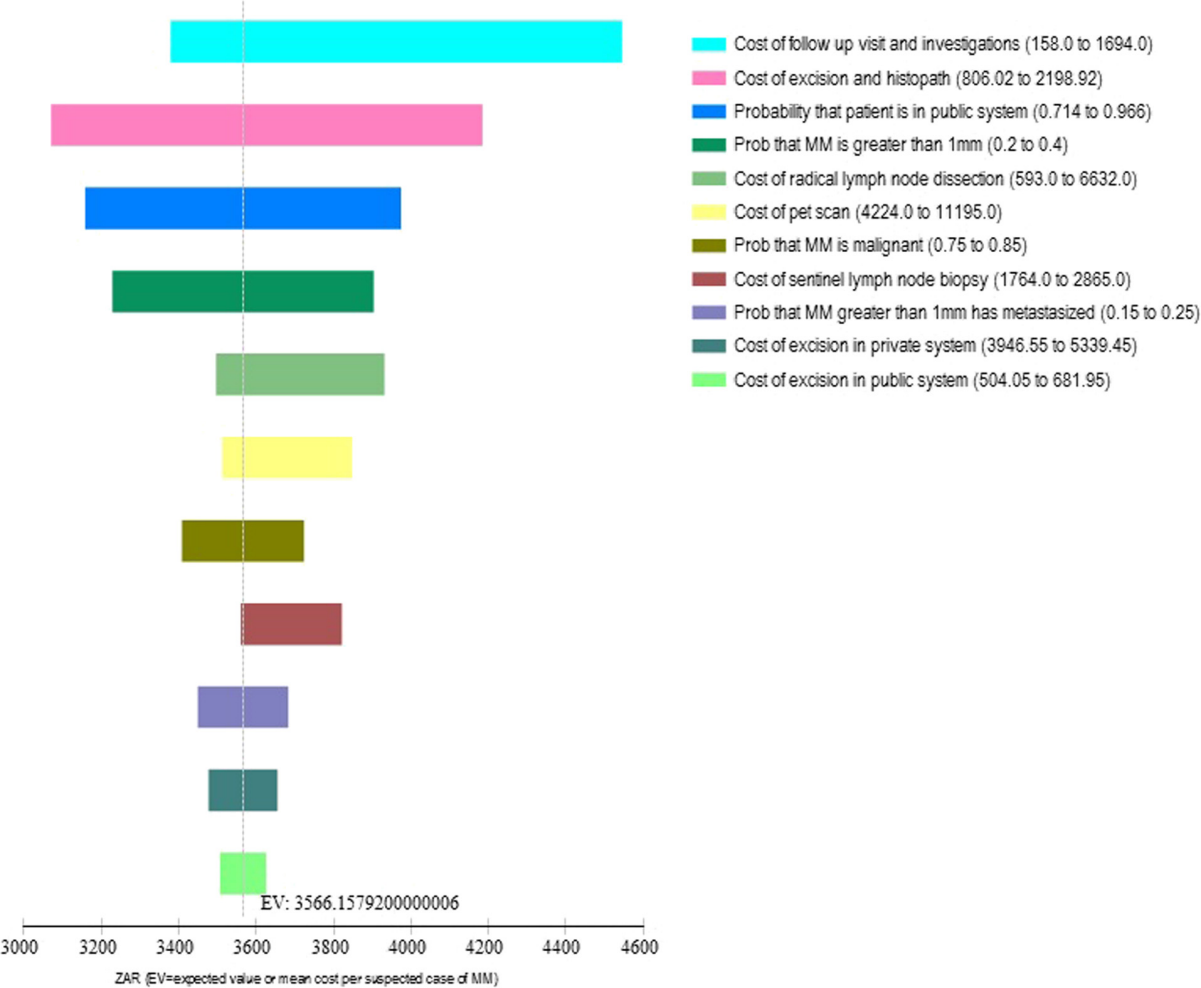
Results of sensitivity analysis for melanoma costs (ZAR)

**Fig. 3:**
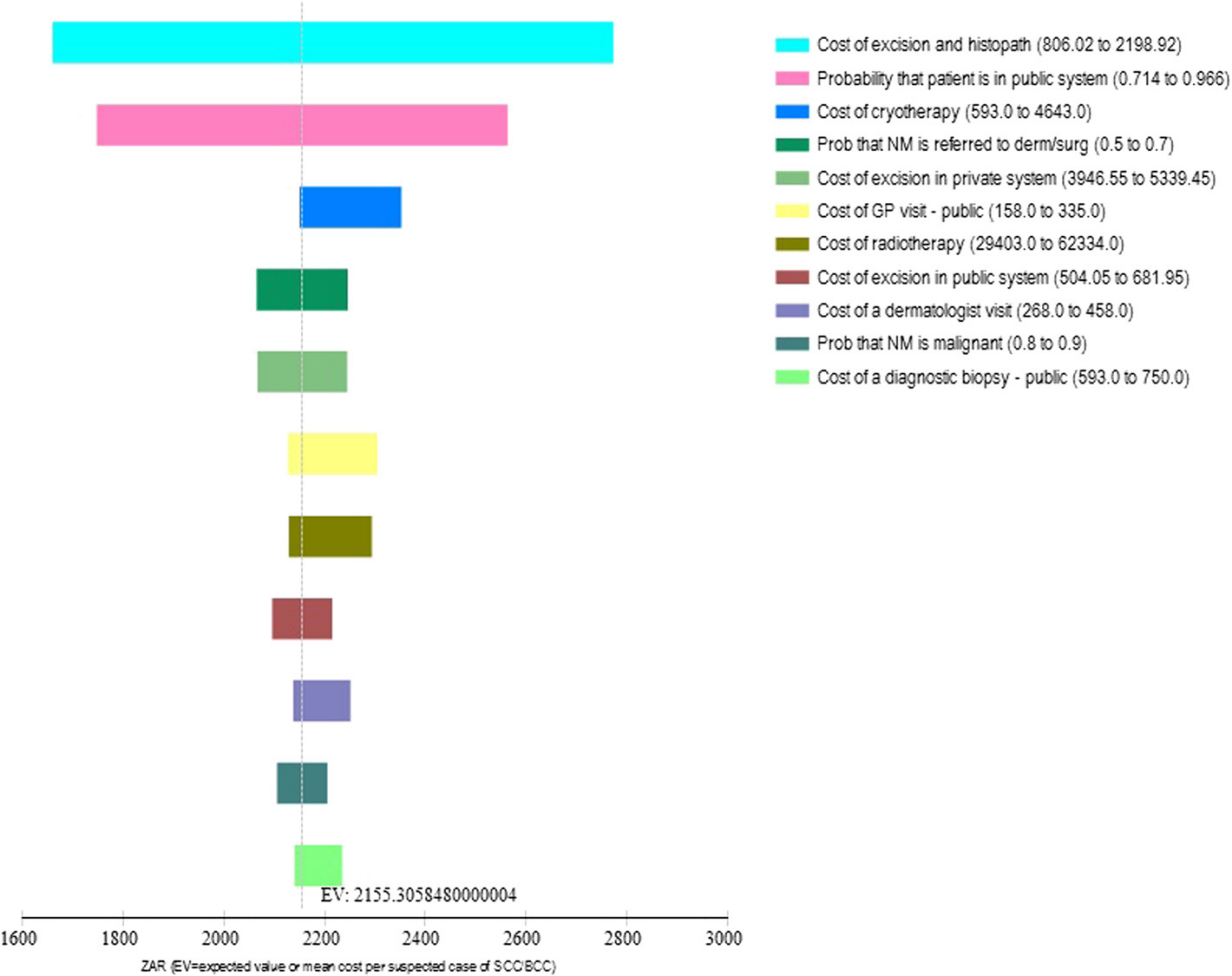
Results of sensitivity analysis for non-melanoma costs (ZAR)

It is standard practice in health economics to acknowledge and transparently quantify the uncertainty present in modelling studies [30]. We undertook one-way sensitivity analyses to assess parameter uncertainty and the tornado figures (Figs. 2 and 3) outline the variables which impact the costing results the most. An additional use of the sensitivity analysis in this study is that it highlights which variables new research could concentrate on to obtain better estimates. Those with high uncertainty and significant influence on the results are displayed at the top of the tornado diagrams (Figs. 2 and 3). They include the costs of follow-up and investigations, cost of excision and histopathology, the proportion of public patients, melanoma size and non-melanoma referrals. Beyond skin cancer, dedicated investment for capturing medical surveillance data in order to create accurate costing models in the South African setting is necessary. This would assist decision-makers in allocating resources where the most public health gain and least costly choices are possible. An efficient health system is one where spending occurs wisely, wastage is eliminated and where the most South Africans can gain better health outcomes.

## Conclusion

In conclusion, this study is a first attempt to provide a snapshot on the financial burden of skin cancer in South Africa. Subject to the caveats herein, the cost of skin cancer in South Africa is substantial and improvements to sun-protection awareness and behaviours are likely to avoid skin cancer development. In doing so, the health of individuals will be improved while also releasing scarce healthcare resources for other pressing public health problems.

## Abbreviations

AIDS: Acquired Immunodeficiency Disease
BCC: basal cell carcinoma
CM: cutaneous melanoma
HIV: human immunodeficiency virus
HPV: Human papilloma virus
NCR: National Cancer Registry
SCC: Squamous cell carcinoma
USD: United States Dollars
ZAR: South African Rand

## Footnotes

1 HPV vaccine was approved in the public sector in South Africa in 2014 (school-based program for girls in Grade 4 in government schools). Vaccines are to be provided to approximately 450,000 girls who will receive a 2 dose vaccine, at a cost of R157 per dose (GSK tender prices reported by MSF). In round 1 of vaccination reached 412,617 girls and round 2 reached 422,000 girls. Authors own calculations based on data included in: Richter, K (2014) Implementation of HPV vaccination in South Africa, https://www.phasa.org.za/implementation-hpv-vaccination-south-africa/ Sans Frontieres (2015). The Right Shot: Bringing Down Barriers to Affordable and Adapted Vaccines, 2nd Edition. January 2015. Downloaded from https://www.msf.org.za/msf-publications/new-msf-report-says-cost-sa-vaccines-too-high.
